# Combined Epiretinal Proliferation and Internal Limiting Membrane Inverted Flap for the Treatment of Large Macular Holes

**DOI:** 10.3390/vision8040063

**Published:** 2024-10-19

**Authors:** Nikolaos Dervenis, Iordanis Vagiakis, Elena P. Papadopoulou, Panagiotis Dervenis, Teresa Sandinha

**Affiliations:** 1 Institute of Ageing and Chronic Disease, University of Liverpool, Liverpool L69 7ZX, UK; 2 Department of Ophthalmology, Medical School, Aristotle University of Thessaloniki, 54636 Thessaloniki, Greece; jvag_@outlook.com (I.V.);; 3 Colchester Eye Centre, Colchester CO4 5JR, UK; 4 St Paul Eye Unit, Royal Liverpool University Hospital, Liverpool L7 8YA, UK

**Keywords:** epiretinal proliferation, macular hole, macular surgery, pars plana vitrectomy

## Abstract

We are presenting a new method for the treatment of large macular holes (MHs) with the use of an inverted flap consisting of both internal limiting membrane (ILM) and epiretinal proliferation (EP). A prospective interventional case series was conducted from September 2021 to January 2023. MH patients with coexistent EP visualized preoperatively in macula optical coherence tomography and with a MHs minimum linear diameter larger than 400 microns underwent standard pars plana vitrectomy with the creation of an inverted petaloid flap (consisting of both ILM and EP) and gas tamponade. Sixteen eyes were included in our case series. MHs closure was successful in all the eyes with a single procedure. The preoperative minimum linear diameter was 707.63 (±164.02 μm), and the preoperative best corrected visual acuity was 1.11 ± 0.52. The postoperative BCVA was 0.51 ± 0.20 (*p* = 0.01) at 6 weeks postoperatively, and the final BCVA was 0.45 ± 0.20 (*p* = 0.008). EP can be safely combined with ILM for the creation of an inverted, petaloid flap to cover and facilitate the closure of large MHs.

## 1. Introduction

Standard macular hole surgery involves pars plana vitrectomy with peeling of the internal limiting membrane (ILM) and gas tamponade with very good anatomic outcomes and satisfactory functional outcomes [1,2]. Postoperative outcomes have been shown to be size-dependent and are worse in larger-size macular holes [3]. To improve the closure rate, different types of flaps and different materials for flap creation have been implemented [4,5]. ILM flaps have the advantage of using a readily available tissue with minimal additional steps compared to standard macular hole surgery and can be used either to cover the macular hole or to fill the hole. Other types of flaps proposed include the epiretinal membrane, retinal autografts, or amniotic membrane [5,6,7].

Epiretinal proliferation (EP) is observed as a mound of homogenous medium reflectivity on the retinal surface [8]. It has been identified in over 10% of macular holes, and its presence has been associated with hole chronicity, size, and the presence of an epiretinal membrane [9]. EP may also be associated with a lower closure rate and worse postoperative functional and structural outcomes [10,11].

We have previously reported the use of combined EP with ILM in a case of a very large macular hole that resulted in hole closure and visual acuity improvement [12]. We describe this technique and the results in a larger series of patients to assess the safety of the technique and the related outcomes.

## 2. Materials and Methods

This study was a prospective interventional case series, and it was conducted according to the Declaration of Helsinki. We included all patients with primary, idiopathic macular holes and a minimum linear diameter > 400 μm operated under a single surgeon firm for the period between September 2021 and January 2023. We excluded patients with recurrent or persistent macular holes, secondary macular holes (e.g., traumatic, myopic), diagnosis of another retinal disease besides macular hole, and previous history of any intraocular surgery besides cataract surgery.

Preoperatively, all patients underwent complete ophthalmic examination, including best corrected visual acuity measurement (BCVA) using the ETDRS charts, intraocular pressure measurement, and slit lamp biomicroscopy, including dilated fundus examination. Imaging with spectral domain optical coherence tomography (OCT) was performed, and the minimum linear diameter was calculated using the OCT software (Zeiss Cirrus 5000, Dublin, CA, USA). The macular holes were graded as large (>400 μm) according to the International Vitreomacular Study Group Classification [13]. Preoperative macular OCTs were reviewed and graded for the presence of epiretinal proliferation.

Follow-up visits were performed 1 day, 2 weeks, 6 weeks, and 3 months postoperatively, and during each visit, the full set of ophthalmic examinations were performed, as recorded above.

The primary study outcome was macular hole closure confirmed by OCT, and the secondary outcomes were BCVA improvement at the end of the follow-up period.

### Surgical Technique

Surgeries were performed with a 23 g pars plana vitrectomy (PPV) setup under subtenon’s anesthesia. Combined phacoemulsification could be performed at the same time if significant lens opacity was identified preoperatively. Posterior vitreous detachment (PVD) was induced using the vitreous cutter on suction mode. The central and peripheral vitreous was removed, and dual blue dye (DORC International, the Netherlands) was used to stain the ILM. Preoperative OCT together with negative stain intraoperatively confirmed the presence of epiretinal proliferation around the macular hole. ILM and EP were peeled centripetally towards the center of the macular hole, in a petaloid fashion, leaving the petaloid flaps attached on a small hinge at the edge of the macular hole. ILM peel could be extended more peripherally in the macular area up to the arcades. An indented retinal periphery search was performed to identify any breaks, and balanced salt solution was exchanged for air initially and then gas on an isovolumetric concentration. The flap could be repositioned to cover and fill the macular hole under air if needed (Video S1). After surgery, all patients were asked to maintain a face down position during the day for 5 days and avoid a supine position when sleeping.

## 3. Results

**Figure 1:**
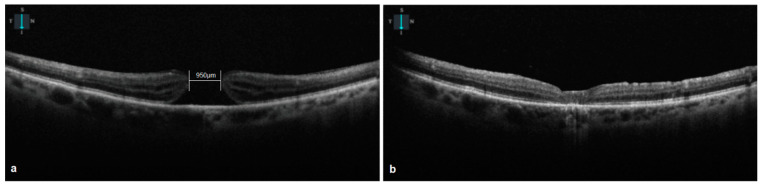
(**a**) Preoperative OCT of a patient with a 950 μm macular hole. (**b**) Postoperative OCT of the same patient. Macular hole closed.

**Figure 2:**
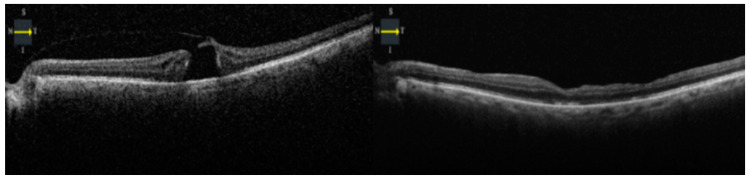
Preoperative and postoperative OCT of a patient showing type 1A closure of the macular hole.

- A total of 16 eyes of 16 patients, 2 male and 14 female, were included in our study. A total of 14 eyes (87.5%) were phakic, whereas 2 (12.5%) were pseudophakic. The mean preoperative BCVA was 1.11 ± 0.52 logMAR, and the mean minimum linear diameter (MLD) of the macular holes included was 707.63 ± 164.02 μm (range 548–950 μm). The mean follow-up period was 6.75 ± 4.40 months. The duration of all MHs was longer than 9 months (accurate duration could not be determined for most patients due to the duration being too long). Table 1 summarizes the baseline patient characteristics.
- Combined phacoemulsification was performed in 12 eyes (75%), whereas 4 (25%) underwent standalone PPV and peel. Posterior vitreous detachment was present in half of the eyes (50%). In the rest of the patients, it was induced intraoperatively. A total of 10 eyes (62.5%) had C2F6 gas tamponade and 6 eyes (37.5%) had C3F8 (Table 2).
- All the macular holes closed postoperatively (Figure 1). Visual acuity improved to 0.51 ± 0.20 logMAR (*p* = 0.01 < 0.05) at 6 weeks and to 0.45 ± 0.25 (*p* = 0.008 < 0.05) at the final follow-up visit. The external limiting membrane (ELM) was continuous in six eyes (37.5%) at 6 weeks postoperatively. At the final follow-up visit, the ellipsoid zone was disrupted in all of the eyes. The closure pattern was 1A for 6 cases (Figure 2) and 1C for 10 cases (Figure 1) at the final follow-up [14]. Table 3 summarizes the surgical outcomes.

## 4. Discussion

Our case series suggests that EP can be safely used as a flap material in combination with ILM to facilitate macular hole closure. There are several advantages of our technique compared to alternative flap techniques which are increasingly used for larger macular holes. Compared to traditional single ILM flap, combination with EP can provide additional tissue volume, which can be used to cover and simultaneously fill the macular hole to facilitate its closure. Moreover, epiretinal proliferation material is considered to be originating from within the macular defect; its composition may be more suitable to be used as a flap, and its preservation during surgery is recommended [15]. Compared to other surgical techniques which only use a part of the perimacular ILM for flapping (thus only a part of the corresponding EP), our technique uses 360 degrees of perimacular ILM and therefore the full volume of the EP. Compared with techniques using tissue outside the macula either autologous (such as retinal autografts, posterior capsule) or allogenic (such as amniotic membrane), our technique provides tissue that, when available, can be easily used to facilitate hole closure with minimal modification to the traditional ILM peeling technique and a short learning curve. There is no need to perform additional traumatic procedures (such as retinectomy to harvest retinal autograft), and there is no need to use material that may not be available (such as posterior capsule), or there might be limitations to its intraocular use (such as amniotic membrane). Even more, considering that EP presence has been associated with macular hole duration, EP is expected to be present in a large percentage of idiopathic macular holes of a larger size as macular hole duration and size are closely related [3]. However, a major limitation of the technique is that it can only be performed in the presence of EP and not in every MH patient.

It can be considered that combined ILM and EP flap acts in a similar way to single ILM flap: it prevents trans-hole fluid flow from the vitreous cavity and forms a scaffold for glial cell migration. However, it needs to be considered that combined, petaloid EP and ILM flap shows increased thickness compared to single ILM flap, and this may facilitate hole filling with flap material and subsequent closure.

There are several limitations of our study, such as its small size and the short follow-up period. Moreover, further prospective studies are needed to analyze and compare additional parameters which might be of interest (such as BCVA, microperimetry, OCT morphology) between the combined flap technique and alternative flap techniques. EP may not be present in all large macular holes, and alternative surgical approaches should be considered in these cases. Further prospective studies are needed to determine the percentage of patients this technique might be applicable to and any potential benefits for macular hole patients.

## 5. Conclusions

Epiretinal proliferation can be safely combined with an internal limiting membrane for the creation of an inverted, petaloid flap to cover and facilitate the closure of large macular holes.

## Figures and Tables

**Table 1:** Baseline patient characteristics [N = 16 eyes].

Characteristic	Summary Measure
	Mean ± SD
Age (years)	70.37 ± 3.16
VA (logMAR)	1.11 ± 0.52
Mean MLD (μm)	707.63 ± 164.02
Mean follow-up (months)	6.75 ± 4.40
Sex	n (%)
Male	2 (12.5)
Female	14 (87.5)
Study eye	n (%)
Right	8 (50)
Left	8 (50)
Lens status	n (%)
Phakic	14 (87.5)
Pseudophakic	2 (12.5)

MLD = minimum linear diameter, VA = visual acuity, SD = standard deviation.

**Table 2:** Surgical technique [N = 16 eyes].

Characteristic	Summary Measure
	n (%)
Combined phacoemulsification	
Yes	12 (75)
No	4 (25)
PVD	
Present	8 (50)
Induced	8 (50)
Tamponade used	
C2F6	10 (62.5)
C3F8	6 (37.5)

PVD = posterior vitreous detachment.

**Table 3:** Surgical outcomes [N = 16 eyes].

Characteristic	Summary Measure
	Mean ± SD
VA at 6 weeks (logMAR)	0.51 ± 0.20
Final VA (logMAR)	0.45 ± 0.25
Macular hole closed	n (%)
Yes	16 (100)
No	0 (0)
ELM at 6 weeks	n (%)
Continuous	6 (37.5)
Disrupted	10 (62.5)
EZ at final follow-up	n (%)
Continuous	0 (0)
Disrupted	16 (100)
Closure pattern at final follow-up	
1A	6 (37.5)
1C	10 (62.5)
ELM = external limiting membrane, EZ = ellipsoid zone, VA = visual acuity, SD = standard deviation	16 (100)

## Data Availability

The data presented in this study are available on request from the corresponding author due to privacy reasons.

## Supplementary Materials

The following supporting information can be downloaded at: https://www.mdpi.com/article/10.3390/vision8040063/s1; Video S1: Surgical video showing the technique of combined epiretinal proliferation and internal limiting membrane flap during macular hole surgery.
